# Research on In Situ Thermophysical Properties Measurement during Heating Processes

**DOI:** 10.3390/nano13010119

**Published:** 2022-12-26

**Authors:** Chenfei Xu, Shen Xu, Zhi Zhang, Huan Lin

**Affiliations:** 1 School of Mechanical and Automotive Engineering, Shanghai University of Engineering Science, Shanghai 201620, China; 2 Mechanical Industrial Key Laboratory of Boiler Low-Carbon Technology, Shanghai University of Engineering Science, Shanghai 201620, China; 3 School of Environmental and Municipal Engineering, Qingdao University of Technology, Qingdao 266033, China

**Keywords:** heating process transient electrothermal technique, thermal conductivity

## Abstract

Biomass pyrolysis is an important way to produce biofuel. It is a chemical reaction process significantly involving heat, in which the heating rate will affect the yield and composition (or quality) of the generated biofuel. Therefore, the heat transfer inside the biomass pellets is important for determining the rate of temperature rise in the pellets. The accurate knowledge of the thermophysical properties of biomass pellets is required to clarify the process and mechanism of heat transfer in the particles and in the reactor. In this work, based on the transient thermoelectric technology, a continuous in situ thermal characterization method for a dynamic heating process is proposed. Multiple thermophysical properties, including thermal conductivity and volumetric heat capacity for corn leaves, are measured simultaneously within a heating process. In temperatures lower than 100 °C, the volumetric heat capacity slightly increases while the thermal conductivity decreases gradually due to the evaporation of water molecules. When the temperature is higher than 100 °C, the organic components in the corn leaves are cracked and carbonized, leading to the increase in the thermal conductivity and the decrease in the volumetric heat capacity against temperature.

## 1. Introduction

Biomass energy is the fourth largest energy source in the world after coal, oil and natural gas in terms of total energy consumption [1], with the dual attributes of being renewable and environmentally friendly. The rapid depletion of fossil fuel reserves makes biomass energy the most promising renewable energy source. Agricultural and forestry residues are the main source of biomass. Many rural areas often burn these agricultural and forestry residues directly as conventional fuels, which are poorly utilized, pollute the environment and endanger human health. Biomass pyrolysis is a key technology for achieving biomass energy utilization [2,3,4]. The process involves rapid heating of biomass under anoxic conditions to obtain high calorific value products, such as oil and natural gas.

During the pyrolysis process, the heating rate of biomass pellets determines the process of the pyrolysis reaction and the quality of the product. Rapid heating can increase the yield of oil and gas products. However, at the same time, the chemical structure in biomass pellets is degraded to form structural defects that hinder the heat transfer inside the biomass pellets, which is an important factor in determining the heating rate [5,6]. The biomass pellets are composed of several biomass material fragments, such as corn leaves, stalks, woods, etc. The in-plane heat conduction of these fragments, rather than the out-of-plane heat transfer among the fragments, contributes to the heat conduction inside the pellets since the loose contact between the fragments will raise a large thermal contact resistance and hinder the heat transfer crossing them. Thus, the in-plane heat conduction is more critical when determining the temperature rise, temperature distribution, and the possible chemical reactions in the fragment. Also, when many researchers [7,8] proposed computational models to elucidate the complexity of biomass pyrolysis, they found that thermal conductivity of the fragments is an important parameter for model building. Obtaining in situ real-time thermophysical properties (thermal conductivity, volumetric heat capacity, etc.) of biomass in pyrolysis reactions has become particularly important to study the heat transfer and temperature dynamic distribution in biomass pellets. Therefore, we endeavor to measure the in-plane thermal conductivity and other thermophysical properties of dried corn leaves undergoing the heating process (mimicking pyrolysis in vacuum).

Various transient methods for quickly measuring thermophysical properties have been successfully developed, including 3ω method [9], microfabricated suspended device method [10], optical heating and electrical thermal sensing (OHETS method) [11] and transient electrothermal (TET) technique [12], etc. These transient methods can measure the thermophysical properties of materials in a very short time and are widely applied to thermal measurement in dynamic processes. Among them, the TET method has the advantages of high measurement accuracy, fast measurement speed and short measurement period, which makes it widely used. It is effective to measure the thermal diffusivity of solid materials (including conductive, semi-conductive or non-conductive one-dimensional structures). The TET method has been used to successfully measure the thermal diffusivity of ultra-thin metal films [13,14], silk [15], DNA fibers [16], graphene [17], etc. The method exhibits the strong measurement ability for measuring the sample of different size ranging from nanometer to millimeter.

In this work, the measurement of the thermophysical properties of dried corn leaves in the heating process by using a continuous in situ thermal characterization method was developed from the TET technique [12]. Furthermore, only a small piece of a corn leaf sample is needed to fulfill thermal measurement and totally exclude the space effect in a packed sample. Section 2 details the mechanism of the TET method and the continuous in situ thermal characterization method for measuring the variation of thermal diffusivity, thermal conductivity, and volumetric heat capacity against temperature. Section 3 describes the verification of the method and investigation on a corn leaf sample and discusses the results.

## 2. Method for Continuous Thermal Properties Measurement for Biomass Particles

### 2.1. Measurement Principle of the TET Method

**Figure 1:**
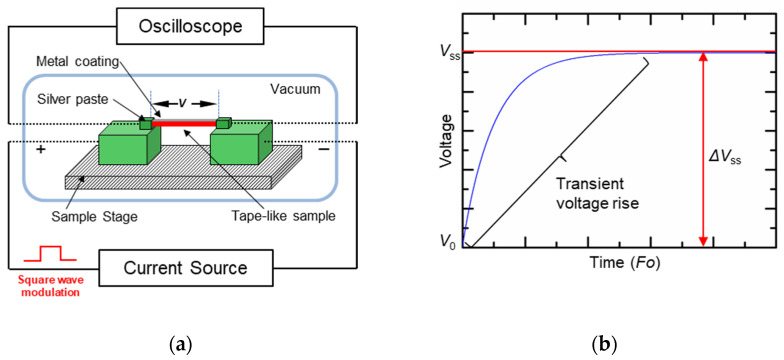
(**a**) Schematic of the experimental principle for the TET technique. (**b**) Voltage response to a step heating current through the sample.

The TET technique utilizes electrical heating to introduce uniform heating over a whole suspended fiber, gathers the transient temperature variation based on voltage/resistance, and determines the in-plane thermophysical properties based on a one-dimensional heat transfer model (Figure 1a) [12]. By applying the TET technique, one can measure the in-plane thermal diffusivity of a tape-like sample with a millimeter length quickly, typically in several seconds. Specifically, the measurement principle is: Before measurement, non-electrical conductive samples should be first coated with a thin metal coating (e.g., iridium (Ir) coating, gold (Au) coating, etc.), so that it can be electrically conductive. A step current then passes through the sample, which causes Joule heating in the metal coating. The temperature rise of the sample can be measured by recording the overall resistance variation of the metal coating, which also works as a temperature sensor based on the effect of temperature on resistance in metal. Because the metal coating is very thin, usually around several tenths of nanometers, the rate of the temperature-rise process of the sample is related to the sample’s thermal diffusivity. For example, when the thermal diffusivity of the sample is small, the temperature raises slowly, so it takes a long time to reach the stable temperature. Applying a constant current after the current is on, the transient temperature rise from the beginning to the thermally steady state is characterized by recording the voltage change at both ends of the sample. Figure 1b shows a theoretical variation of the voltage between both ends of the sample with time in the TET measurement. In most cases, metal has a constant resistance–temperature coefficient, and thus, one can monitor the voltage change of the sample to measure its temperature change. By fitting the normalized average temperature rise of the sample according to the variation of the voltage, the thermal diffusivity of the sample can be obtained. 

### 2.2. One-Dimensional Heat Transfer Model

Since the length of the tape-like sample is much larger than its diameter, a one-dimensional heat transfer model along the axial direction (*x* direction) can be satisfied, and the heat transfer equation along the *x* direction can be expressed as Equation (1):

(1) $$\frac{\partial \left(\rho c_{p}T\right)}{\partial t}=k\frac{\partial^{2}T}{\partial x^{2}}+q_{0},$$

where *ρ*, *c*_p_ and *k* are the density, specific heat, and thermal conductivity of the sample. *T* is the sample temperature. The effect of thermal convection is eliminated by employing a vacuum environment, and the radiation effect is included in the conduction properties and will be discussed and excluded later. 

The initial condition is:

(2) $$T\left(x,t=0\right)=T_{0},$$

and the boundary conditions are:

(3) $$T\left(x=0,t\right)=T\left(x=L,t\right)=T_{0},$$

where *T*_0_ and *L* are the room temperature and the length of the sample.

By solving Equation (1) using Green’s function, the relationship between the normalized temperature rise θ* [θ* = (*T* − *T*_0_)/(*T*_ss_ − *T*_0_)] of the sample and time *t* can be obtained as shown in Equation (4):

(4) $$T^{*}=\frac{96}{\pi^{4}}\sum_{m=1}^{\infty }\frac{1-\exp\left[-{\left(2m-1\right)}^{2}\pi^{2}\alpha t/l^{2}\right]}{{\left(2m-1\right)}^{4}},$$

The TET thermophysical property measurement is carried out in a vacuum environment with a vacuum degree of 2 × 10^−3^ mbar, so the influence of convective heat transfer can be safely ignored. In addition, the thermal radiation between the tested sample and the environment and the metal coating on the non-conductive sample will affect the accuracy of the thermal diffusivity measurement. For details, please refer to literature [12,17].

The contribution to the measured thermal diffusivity from the thermal radiation is denoted by *α*_rad_ and can be expressed as Equation (5):

(5) $$\alpha_{rad}=\frac{1}{\rho c_{p}}\frac{8\varepsilon_{r}\sigma T_{0}^{3}}{D}\frac{L^{2}}{\pi^{2}},$$

where *ε*_r_ is the effective emissivity of the sample, and *σ* = 5.67 × 10^−8^ W m^−2^ K^−4^ is the Stefan–Boltzmann constant. The contribution caused by the metal coating, *α*_coating_, can be expressed as:

(6) $$\alpha_{coating}=\frac{L_{Lorenz}T\cdot L}{RA_{W}\rho c_{p}},$$

where *A*_w_ is the cross-sectional area of the sample before being coated; *L*_Lorenz_ is the Lorenz number for the metal coating; and *R* is the electrical resistance of the sample. After removing the influence of *α*_rad_ and *α*_coating_ from the measured thermal diffusivity, the actual thermal diffusivity *α*_real_ of the sample can be obtained as *α*_real_ = *α*_real_ − *α*_rad_ − *α*_coating_.

Though the transient temperature rise contributes to the determination of the thermal diffusivity, the temperature increase at the thermal steady state can help to calculate the thermal conductivity directly, without the knowledge of other thermal properties, like specific heat. According to Equation (4), when *t* goes to infinite, the steady-state temperature has an expression as *T*_SS_ = *T*_0_ + *q*_0_*l*^2^/12*k*. One can determine the thermal conductivity *k* immediately with Equation (7):

(7) $$k=I^{2}RL\times \left(dR/dT\right)/\left(12A_{W}\times \Delta R\right),$$

where *I* is the current; d*R*/d*T* is the electrical resistance–temperature coefficient of the metal coating; and Δ*R* is the change of the electrical resistance of the sample during Joule heating. Furthermore, from the definition of thermal diffusivity *α*: *α* = *k*/*ρ·c*_p_, the volume- specific heat *ρ·c*_p_ of the sample can then be obtained. That is, the temperature *T*, thermal diffusivity α, thermal conductivity k and volumetric specific heat *ρ·c*_p_ of the sample can be obtained simultaneously in a single square wave period of the measurement. 

### 2.3. Continuous Thermal Characterization Based on the TET

#### 2.3.1. Mechanism of the Continuous in Situ Thermal Characterization

**Figure 2:**
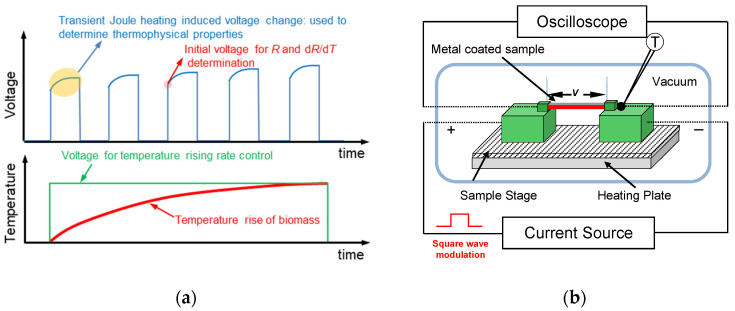
(**a**) Mechanism of the continuous in situ thermal characterization method. (**b**) A lab-developed, experimental setup for continuous thermal characterization.

One important parameter in the TET method is the characterization time. From Equation (4), it is clear that the normalized temperature rise θ* mainly depends on *αt*^2^/*L*, which is a dimensionless parameter, Fourier number (*Fo*). Then, the characteristic time *t*_c_ has been defined when *Fo* equals to 0.2026. *t*_c_ (=$\sqrt{0.2026L^{2}/\alpha }$) indicates the spent time when the temperature of a specific sample (having a specific thermal diffusivity *α*) almost reaches the thermal steady-state temperature after being heated. Thus, one can estimate the possible time that one Joule heating and measurement may take. It depends on the thermal diffusivity and length of the sample, and in turn, *t*_c_ (as well as the TET measuring time) can be adjusted by selecting the desired length of the sample. Based on this mechanism, we cut samples into the suitable length, and the time for one TET measurement would be much shorter than the heating process, as shown in Figure 2a. 

#### 2.3.2. A typical Experimental Setup

Figure 2b shows a typical experimental setup. The tape-like sample is fixed on a lab-made sample stage. It is suspended between two electrodes, which are placed on a slide of glass. Then, the sample stage is placed on a heating plate. The heating plate is provided with a controllable heating source to generate a slow heating process. The temperature-rise rate can be adjusted by the power source connected to the heating plate. On the sample, another modulated square-wave current is fed to generate ‘fast’ Joule heating and a small temperature increase in the sample to acquire the thermal diffusivity of the sample. The oscilloscope is attached to the two ends of the sample and records the fast voltage variation due to Joule heating. A thermocouple is attached to the one electrode and close to the sample end to monitor the real-time temperature of the sample. It is necessary for calculating d*R*/d*T* of the nanometer thick coating, since the electrical properties of the coating may be greatly different from its bulk counterpart.

## 3. Results and Discussions

### 3.1. Verification of the Method and Experimental Setup

First, an ultrahigh, molecular-weight, polyethylene (UHMW-PE) fiber is employed to verify the method and experimental setup. UHMW-PE fibers have a high crystalline content, >90%, so they work well in heat conduction along its axial direction. In previous studies, the similar fibers were investigated at the room temperature and reported to have a high thermal diffusivity of 1.1 × 10^−5^ m^2^/s. According to this high thermal diffusivity and the definition of *Fo* (=0.2026) mentioned above, a one-millimeter fiber can almost reach the new thermal steady state in 0.02 s after Joule heating begins. In other words, the measurement duration is only about 0.02 s. Thus, we could set a heating process from room temperature to 90 °C with a time span of 200 s. During the 0.02 s- measurement duration, the temperature rise induced by the heating stage will be as low as 6.5 × 10^−3^ °C. It is safe to neglect the heating stage raised temperature rise and directly record the voltage variation and the temperature rise through the thermocouple to determine the thermophysical properties change against time. 

**Figure 3:**
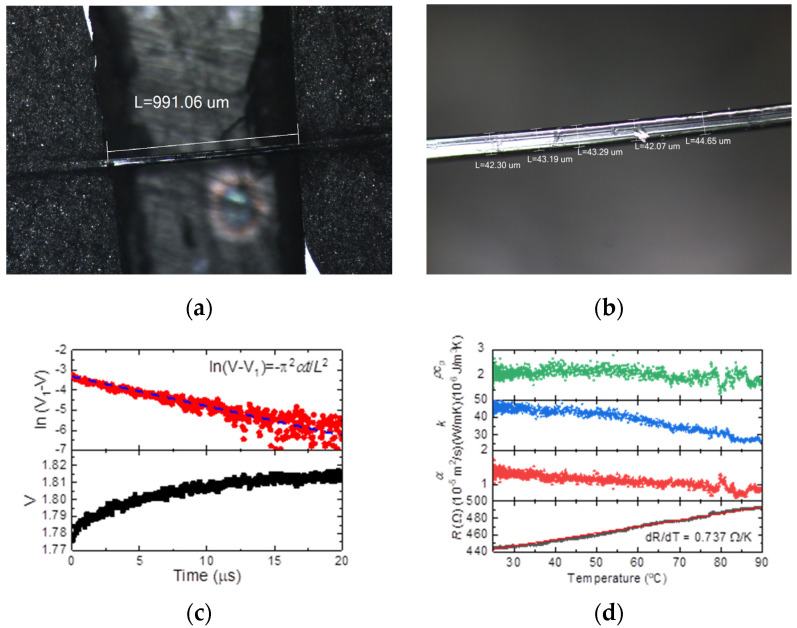
Verification of the method by using a UHMW-PE fiber. (**a**) Length and (**b**) width of the sample UHMW-PE fiber. (**c**) A typical measured voltage variation (bottom) and thermal diffusivity determination (top). (**d**) The variations of thermal diffusivity *α*, thermal conductivity *k*, volumetric heat capacity *ρ*·*c*_p_, and electrical resistance *R* for the UHMW-PE fiber against temperature.

We prepare a thin UHMW-PE fiber as a standard sample, which is about 909.5 μm long and 43.2 μm width, as shown in Figure 3a,b. It has been coated with 20 nm Ir film and fixed on a sample stage. Silver paste is applied to its two ends to guarantee a good electrical and thermal connection between the sample end and the electrode. In the measurement, the Joule heating current is 4 mA and modulation frequency is 10 Hz. The room temperature is around 23 °C. Figure 3c shows a typical voltage rising curve against time (bottom panel) and data fitting [18] (top panel) at a certain temperature during the Joule heating. The determined thermal diffusivity is 1.13 ± 0.03 m^2^/s. The variation of thermal diffusivity for the UHMW-PE fiber is shown in Figure 3d. It linearly decreases against temperature. 

Furthermore, it is important to note that we can obtain the electrical resistance of the sample at the very beginning of Joule heating, since the current is constant. That means the real temperature of the sample can be derived through this electrical resistance, and the relationship between the electrical resistance of the Ir coating against time can be recorded. As shown in the bottom panel in Figure 3d, the resistance of the coating increases linearly against temperature. The electrical resistance–temperature coefficient (d*R*/d*T*) is determined to be 0.737 Ω/°C. With the obtained thermal diffusivity and d*R*/d*T*, we can calculate the thermal conductivity and volumetric heat capacity, as shown in Figure 3d. The thermal conductivity decreases linearly against the rising temperature, and the volumetric heat capacity keeps almost constant in this temperature range. When the temperature is above 90 °C, the fiber begins to melt, and the coating is broken at the same time, which causes the loss of the voltage signal. However, before the melting of fiber, the measured thermal properties agree well with the value and the varying trend reported in previous work [19]. It verifies this method that the experimental setup works well for thermal characterization during the heating process.

### 3.2. Thermal Characterization for Corn Leaves during Heating

To investigate the thermophysical properties’ variation in heating processes, a corn leaf is used as a typical biomass sample. The corn leaf is freeze-dried and cut into tape-like shape. Then, a layer of Ir film (40 nm thick) is coated on the leaf surface to make it conductive, and its two ends are fixed on the electrode with the silver paste as that in the UHMW-PE fiber sample preparation. However, obviously different from the UHMW-PE fiber, the corn leaf fiber has a low thermal conductivity/diffusivity. It needs more time to reach the criteria of *Fo* = 0.2026. To ensure that the heating stage temperature rise is negligible in the Joule heating duration, the sample is prepared into a short length, and it can quickly reach the thermal steady state during one Joule heating and measurement duration. The length of the corn leaf sample prepared as shown in Figure 3 is about 0.65 mm, and the time required for the sample to reach the thermal steady state is about 0.85 s. Different from the above, the UHMW-PE fiber having a round cross-section, the corn leaf is a flat film with a width of 0.45 mm. Its length is much larger than its thickness (less than 0.06 mm), and the time duration for Joule heat to propagate in the thickness direction and to reach, a thermal steady state (~0.02 s) will be much shorter than that required to propagate along the length and reach a thermal steady state (~0.85 s). Therefore, when applying the TET method to measure the corn leaf sample, it is reasonably assumed that the temperature is uniform along the thickness direction, and Joule heat mainly transfers along the length of the sample, which agrees well with the one-dimensional heat transfer model.

**Figure 4:**
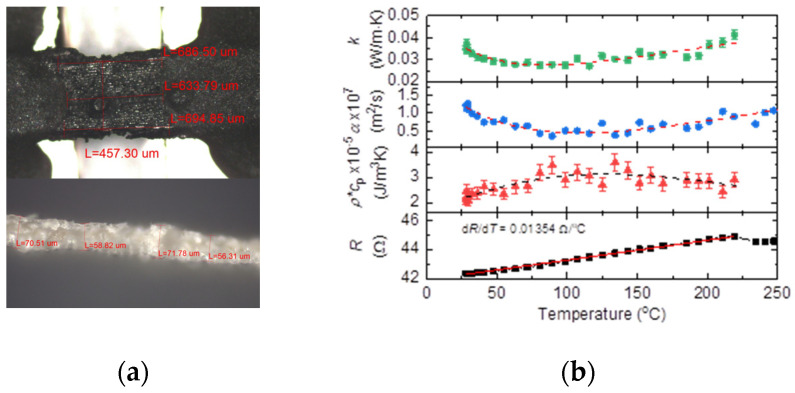
Continuous thermal characterization of corn leaves. (**a**) Length (top), width (top), and thickness (bottom) of the corn leaf sample with Ir coating. (**b**) The variations of thermal diffusivity *α*, thermal conductivity *k*, volumetric heat capacity *ρ*·*c*_p_, and electric resistance *R* for the corn leaf sample against temperature. The dash lines show the varying of the properties.

Under the condition of a heating rate of 0.87 °C/s, the measured thermal diffusivity of corn leaves is shown in Figure 4. At room temperature, the thermal diffusivity of corn is about 1.2 × 10^−7^ m^2^/s, which is similar to the thermal diffusivity of biomass materials reported in the literature [20,21]. As the temperature increases, the variation of the thermal diffusivity of the corn leaf sample shows a trend of first decline and then rise, and reaches the lowest value near 110 °C. The electrical resistance–temperature coefficient of the 40 nm Ir coating on top of the corn leaf shows a good linearity in the temperature range from room temperature to 225 °C. Below 225 °C, d*R*/d*T* is fitted to be 0.01345 Ω/°C, while above 225 °C, the Ir coating may be broken by the chemical reaction in the corn leaf, and the electrical resistance becomes unstable. The value of d*R*/d*T* is abnormal, and the thermal conductivity and volumetric heat capacity cannot be defined then. The thermal conductivity *k* of the sample can be obtained below 225 °C, as shown in Figure 4b. The measured *k* here is lower than that reported in other literature. This is because the sample is dried in advance and is measured in the vacuum, where different environmental conditions may cause the lower *k* than others [22,23,24]. *k* has the same varying trend as that of the thermal diffusivity. When the temperature is lower than 100 °C, it decreases with the increase in temperature, which is mainly due to the small amount of water remaining in the corn leaf samples. Because the thermal conductivity of water is much higher than the organic components in corn leaves, heating and evaporation of water molecules reduces the overall thermal conductivity of the sample corn leaf. As the temperature further rises, the organic matter in the corn leaves is cracked, the carbonization gradually occurs at a lower heating rate, and the carbon in the solid residue increases. The thermal conductivity of carbon is higher than the organic components in corn leaves (including cellulose, hemicellulose, lignin, etc.), so the increment in carbon content will increase the thermal conductivity of the entire solid phase residue. Therefore, the overall *k* of the sample corn leaf increases [25]. 

Then the volumetric heat capacity of the corn leaf is calculated by using α= *k*/*ρ*·*c*_p_, and the result is shown in Figure 4b. *ρ*·*c*_p_ first rises against temperature, and after the temperature is higher than 120 °C, it declines. Volatilization of a small amount of water has little effect on the density of solid-phase components in the initial heating stage, and *ρ*·*c*_p_ shows a slight increasing trend against temperature. When the temperature is higher than 100 °C, due to carbonization of bio-organic molecules, small gas molecules are generated and leave the solid structure, which produces a loose porous structure in solid, reduces the density of the solid phase components, and results in a decrease in *ρ*·*c*_p_ with temperature.

## 4. Conclusions

In this work, we have developed a continuous in situ thermal characterization method and established a corresponding experimental setup for the continuous thermal diffusivity, thermal conductivity, and volumetric heat capacity measurement for UHMW-PE fibers and corn leaves during heating processes. The verification by using the UHMW-PE fiber showed a good measurement ability of the method and the experimental setup. Furthermore, by applying the new method, the thermophysical properties’ variation of corn leaves has been investigated in a slow heating process. In a vacuum, the thermal diffusivity first decreased from 1.2 × 10^−7^ m^2^/s to 0.5 × 10^−7^ m^2^/s below 100 °C and then increased to 1.2 × 10^−7^ m^2^/s until the coating broke. The thermal conductivity showed the similar varying trend as the thermal diffusivity. It decreased from 0.035 W/m·K to 0.028 W/m·K and increased to 0.04 W/m·K. The volumetric heat capacity changed inversely. It increased first from 2.2 × 10^5^ J/m^3^K to 3.0 × 10^5^ J/m^3^K and then decreased to around 2.2 × 10^5^ J/m^3^K. Both dehydration and carbonization on the thermophysical properties of biomass materials contributed to these variations. However, the time resolution for the measurement of corn leaves was somehow limited by the sample size/characteristic time. In a sample with a much smaller size, further work could be conducted on faster heating processes, such as fast pyrolysis, in the future.

## Data Availability

The data presented in this study are available on request from the corresponding author.
